# Reasoning about “Capability”: Wild Robins Respond to Limb Visibility in Humans

**DOI:** 10.3390/bs6030015

**Published:** 2016-07-21

**Authors:** Alexis Garland, Jason Low

**Affiliations:** 1 Institute of Cognitive Neuroscience, Department of Biopsychology, Ruhr University Bochum, Bochum 44801, Germany; 2 School of Psychology, Victoria University of Wellington, Wellington 6140, New Zealand; Jason.Low@vuw.ac.nz

**Keywords:** capability, physical causality, intentional actions, New Zealand robin, pilfering

## Abstract

Little comparative work has focused on what nonhumans understand about what physical acts others are capable of performing, and none has yet done so in the wild, or within a competitive framework. This study shows that North Island robins visually attend to human limbs in the context of determining who to steal food from. We presented 24 wild North Island Robins (*Petroica longipes*) with two experimenters. Robins could choose to steal a mealworm from one of two experimenters: one whose limbs were exposed and one who underwent a range of visual obstructions in two experiments. In most conditions, robins preferred to steal food located near the experimenter whose limbs were obscured by a cloth or board rather than food located near the experimenter whose limbs were not obscured. The robins’ responses indicate that human limb visibility is associated with reduced access to food. Current findings lay the groundwork for a closer look at the potential general use of causal reasoning in an inter-specific context of using limbs to perform physical acts, specifically within the context of pilfering. This study presents one of the first tests of the role of visual attendance of potential limb availability in a competitive context, and could provide an alternative hypothesis for how other species have passed tests designed to examine what individuals understand about the physical acts others are capable of performing.

## 1. Introduction

The ability to reason about agency and action [1] has been explored in a myriad of ways and species. Species from newly-hatched chicks to chimpanzees are sensitive to what their predators or competitors can and cannot see [2,3,4], goal-directed action [5,6,7,8] and gaze direction [9,10,11,12,13], and even the availability of causality-relevant information, such as tool availability [6,14].

The role of the limbs in the execution of specific actions is linked to physical causality and has been studied in chimpanzees [15], but not yet in avians. For both primates and avians, this may be an important capacity for reasoning about the actions of conspecifics and actions; affecting success in competing for mates, foraging, defense of food stores, and possibly assessing strength and weakness in competitors, predator and prey alike. For North Island robins in particular, it begs the question of whether there exists a basic understanding of physical causality as it relates to limb mobility in humans; a species not only much more taxonomically distant than humans and other primates, but one with which exposure on an evolutionary scale is only extremely recent. There are also clearly much greater morphological differences between songbirds and humans than between chimpanzees and humans. To this end, this experiment was adapted to test wild robins’ abilities to track salient observable cues—visibility of arms and legs—to potentially predict basic “capability” (the ability to perform specific tasks) in humans. This paper examines whether North Island robins visually attend to human limbs in the context of determining who to steal food from. We present here one of the first tests of the role of visual attendance of potential limb availability in a competitive, rather than cooperative, context. This may provide an alternative hypothesis for how other species have passed tests designed to examine what individuals understand about the physical acts others are capable of performing. Examining a potential link between limb availability and theft preference and avoidance, we begin to examine the role this connection may play in inter-specific decision-making about foraging risk, intention, and perception of physical capability between taxonomically- and evolutionarily-distant species.

The majority of studies looking at perception of capability in a human experimenter by another species examines the problem in terms of mental state, examining intentionality or goal directedness of human experimenters. Whereas chimpanzees did not preferentially distinguish human experimenters who accidentally or deliberately failed to offer food [16], both chimpanzees and human infants discriminated between “unwilling” and “unable” experimenters [5,6], as did capuchin monkeys [8]. A recent study suggests that humans and chimpanzees anticipate goal-directed outcomes, but scan the actions differently—humans attend to others’ faces reliably more than do chimpanzees [17]. Outside of primates, jackdaws [18], domestic dogs [19,20], dingoes [21], dolphins [22], goats [23], and horses [24] all appear to respond in different contexts with varying levels of success to human social cues and gestures, such as pointing. In some of these species, such as goats, horses, and jackdaws, domestication and/or commensalism may have played a role in the evolutionary and historical development of attendance to important human social cues.

A recent study with African grey parrots (*Psittacus erithacus*) [25] found that parrots displayed more requesting behaviour when an experimenter was unwilling to give them seeds—and that their behaviour pattern changed according to attention. However, African grey parrots were unable to use pointing gestures or gaze consistently as cues [26]. Jackdaws, in contrast, are able to use pointing as a cue, but only by a familiar caretaker [18]. Despite such evidence, little has been done to investigate the more obvious and preliminary question of whether other species respond simply to salient observable cues—such as visibility of limbs—to predict a human experimenter’s physical capability, regardless of motivational state. As Vonk and Subiaul [15] point out, such a task does not require the subject to reason about unobservable mental states, or make the inferences necessary to understand a complex gesture, such as pointing or subtle mental state differences, like “unwilling”. The present study focuses on presenting experimenters who appear physically, rather than motivationally, incapable. The amount of occlusion of each experimenters’ body varied such that barriers either did or did not impede movement of the limbs towards mealworm prey.

**Figure 1:**
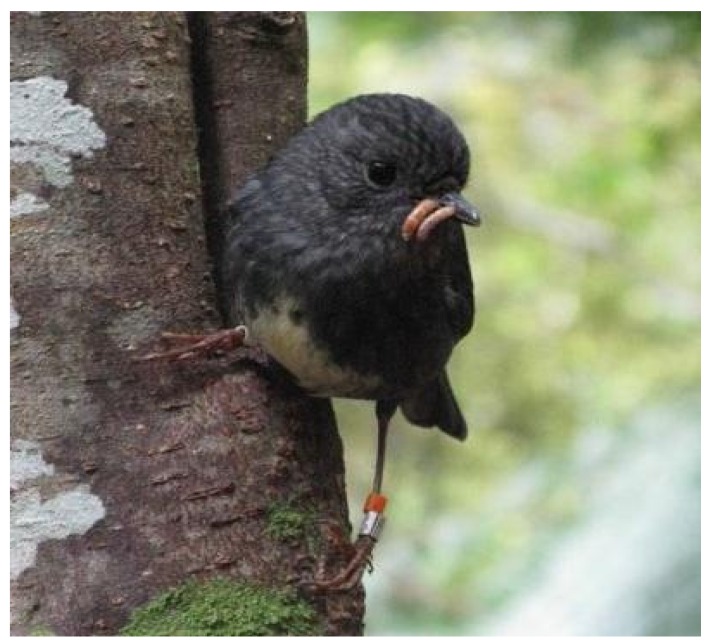
A banded robin with mealworm prey.

In this study, we asked whether wild North Island robins (Figure 1), a species endemic to New Zealand, were able to reason about physical capability in humans. To do so, ten different conditions were presented to 24 adult robins in two experiments, in which they were given the opportunity to “steal” a mealworm from one of two human competitors. If robins’ behaviour in competitive foraging or pilfering opportunities takes human capability into account, subjects should opportunistically steal from the human experimenter whose body or limbs are more occluded. In addition to successfully responding to body and limb visibility to make contextually-appropriate decisions, robins must also be able to spontaneously utilize information about movement and physical access in multiple mammalian experimenters to make a task-relevant decision.

The methodology adopted for this study is modelled after that of Vonk and Subiaul’s [15] chimpanzee study on capability, but was adapted both for the use of wild birds as subjects, and conducting trials out in the field. In addition to either displaying or obscuring arms and legs, the appearance of the nose/mouth area of the face was also altered. Use of the beak is frequent in territorial defence, pilfering, and the majority of other interaction with and manipulation of the environment around them; for a bird it is as much of a “limb” as other appendages; jackdaws for example, use beak pointing to direct each other’s attention to objects [18]. As such, the nose/mouth area of human experimenters were also occluded or displayed as a potential analogue to an avian beak. Most importantly, this choice task is framed within a competitive paradigm. Instead of being asked to choose a food item near a human who will cooperate by providing that item at request when physically possible, robins are asked to choose between stealing the food item from an experimenter, given differing observable physical cues. In the present study, various conditions are presented in which one experimenter is occluded such that it is more difficult for her to use her limbs to access the mealworm in front of her. An additional significant feature is the static location of the mealworm bait in reference to the experimenters. The present study does not attempt to address robins’ ability to flexibly apply an awareness of causality or capability, in that the position of the mealworm was not altered in order to appear within reach of only one limb region or another between conditions. It does, however, ask whether a basic understanding of human limb capability in cache defence is present in a species that has not co-evolved with humans.

This study intends to examine whether robins respond to a competitive food-pilfering task by utilizing information about the visibility of human limbs. If robins prefer to pilfer food prey from humans with occluded limbs, when barriers visibly impede limb movement towards presented prey, then this would indicate that they associate human limb visibility with reduced access to food.

## 2. Methods

### 2.1. Study Area and Subjects

Wild North Island robins (*Petroica longipes*) were the focus of this study. A total of 24 adult robins participated in this study. Each individual robin was identified by a unique combination of coloured bands around their legs (see Figure 1). Subjects in this study were banded under the initiative of the Zealandia sanctuary staff by volunteers across a period of years prior to the study itself, either as adults with a spring-loaded clap trap, or as nestlings via temporary removal by hand. Data presented here was collected between November 2011 and March 2012. Some subjects were known to have successfully participated in prior behavioural experiments and have regular exposure to visitors and staff walking through the sanctuary forest paths transecting the territories. Robins are not provided with food outside experimentation, and hunt freely for insects in the leaf litter within their self-established defended territories. North Island Robins (*Petroica longipes*) face a highly competitive foraging paradigm [27] and practice food hoarding [28] which has a high rate of pilfering [29]. Robin pairs compete for foraging space by constantly defending established territories (and food hoards) from neighbouring pairs. Subjects appear highly motivated to pilfer prey presented by human experimenters.

### 2.2. Procedure

Two experimenters performed each experiment, both acting as the competitors (Experimenter One and Two, E1 and E2) presenting mealworms (*Tenebrio molitar*) on square wooden platforms. Each platform was constructed with identical shallow circular indentations and lined in plastic, to prevent immediate escape of the mealworm prey. The female experimenters were both of similar appearance and build, wore the same clothing (black rain pants, long-sleeved green shirt, black shoe covers) intended both to function in the field environment and avoid colours that may create potential unintended signalling (such as white, similar to their frontal spot) [30]. One experimenter gave verbal instruction ahead of each trial, and movements were synchronised during each trial. Condition positions and verbal instructions were counterbalanced between experimenters.

Subjects were located on known established territories, in which each set of trials was conducted. Clapping and whistling was used to gain the subject’s attention at an appropriate location within the territory ahead of each test session. For video recording, a camera was set up on a flexible field tripod, attached to trees or other landscape features as close as possible to a 90 degree side view of both the subject and experimenters, varying due to the slope of the valley forest landscape and vegetation density.

Both experimenters approached the subject from the front, each holding a wooden platform and a single mealworm. Each platform was within easy reach of only the experimenter directly behind it. At approximately 1–2 m away from the subject, experimenters squatted and set their platform on the ground approximately 0.3 m in front themselves. The robin observed from a position approximately equidistant between E1 and E2 and their platforms. Continuing to synchronize movements, experimenters then held their mealworms out at waist height (approximate 40 cm) over the platform, for 10–20 s (visible, pinched between two fingers) until the robin was attending. Experimenters then dropped the mealworms simultaneously onto the platform and immediately assumed the final position described for each condition below. In each condition, one experimenter’s limbs was occluded such that prey was less easily reached than the other experimenter. The robin subject was then given the opportunity to approach one of the two competitors, retrieving one of the two mealworms. After retrieval, experimenters both resumed forward positions and picked up their respective platforms.

In order to avoid aversion or startling due to sound or movement while assuming positions at such close proximity, both experimenters made similar limb and body movements for the same length of time, regardless of the final position. Outcomes were recorded in situ as well as with a video camera, as a precaution. Responses were verified by both experimenters and always unambiguous. Statistical analysis of results was performed by conducting a binomial test on each condition in order to examine statistical significance in the choice of pilfering from E1 or E2 across the 24 subjects in each of the 10 conditions (six in the first experiment and four in the second experiment).

## 3. Experiment 1

### 3.1. Procedure

Twenty-four adult robins were exposed to each of six conditions, using Latin Squares (in this case, a 6 × 6 array with each condition occurring only once in each row and column) in order to alternate the order of exposure, experimenter side (left and right), and experimenter position (visual access or none) across the 24 robins. Opaque brown cloth was used measuring 210 cm by 154 cm to obscure the entire body or limbs of either experimenter. The same type of cloth was used for obscuring part of the face, measuring 26 cm by 75 cm. Each bird was exposed to each condition once.

**Figure 2:**
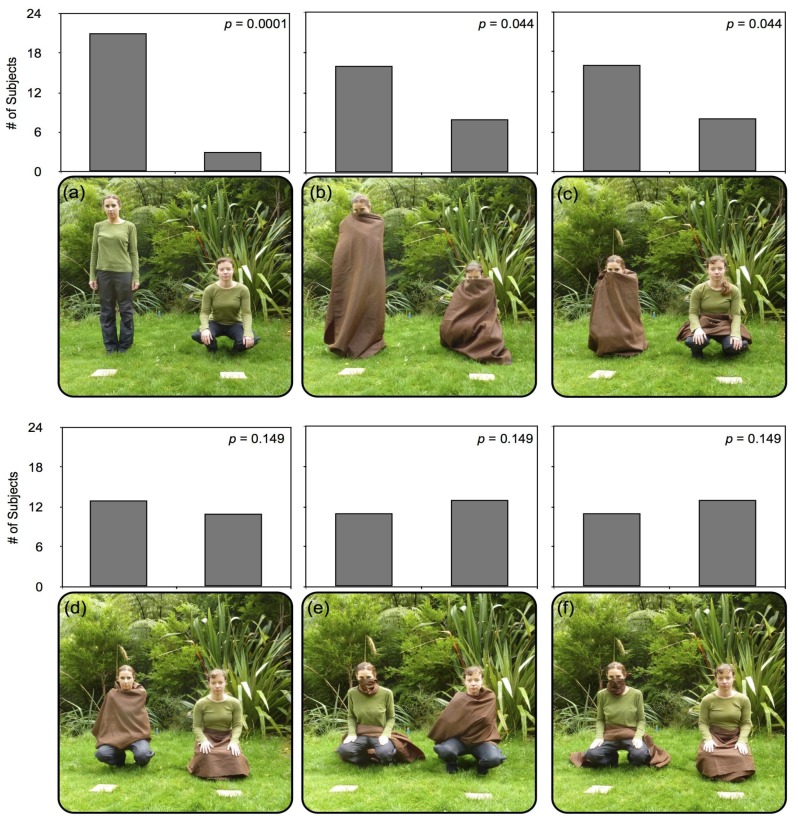
The six conditions presented to robins in Experiment 1, using opaque brown cloth to obscure the body or limbs of the experimenters (E1 and E2). Results for all six conditions in Experiment 1, displaying number of subjects pilfering from each experimenter, with one trial per condition per subject. In condition 1 (**a**) experimenters are both uncovered, while E1 stands and E2 squats; where in condition 2 (**b**) both are covered, with E1 standing and E2 squatting; In conditions 3–6 (**c–f**), both experimenters squat; Condition 3 (**c**) presents E1 entirely covered, while every limb region is visible for E2, with the cloth draped across her lap; In condition 4 (**d**), E1’s arms are obscured, while E2’s legs are covered; Condition 5 (**e**) presents E1 with only her nose/mouth hidden and a cloth draped across her lap, but E2’s arms are obscured; In condition 6 (**f**), E1’s nose/mouth is covered, but legs and arms are visible (cloth draped across lap), and E2’s legs are hidden.

Condition 1. (See Figure 2a) Neither experimenter was covered in this condition. Subjects were presented with a choice between Experimenter One (E1) in the standing position, and Experimenter Two (E2) in the squatting position. Arms, legs, and faces were all fully visible in this condition, but the face and arms of E2 are no longer within easy reach of the mealworm platform.

Condition 2. (See Figure 2b) Both experimenters were covered in this condition, revealing only their head from just above the bridge of the nose. E1 stood, covered in opaque brown cloth, while E2 squatted, covered in identical opaque brown cloth. Arms, legs, and faces were all fully occluded in this condition, but the eyes and facial region of E1 was more distant from the platform in standing position.

Condition 3. (See Figure 2c) E1 was covered in opaque brown cloth, with her entire body visually occluded from the bridge of the nose to the ground. E2 was not covered, but draped an identical brown cloth over her lap, without occluding any limbs or a significant portion of her body. Both experimenters squatted in the final position. The arms, legs, and face of E2 were all fully visible and within easy reach of the platform, whereas E1 could only do so with difficulty, after removing the cloth from any of these regions.

Condition 4. (See Figure 2d) E1 covered her arms with an identical brown cloth, occluding her arms, torso and shoulders. E2 covered her legs with an opaque brown cloth, visually occluding her legs from the waist down. Both experimenters squatted in the final position. Both experimenter’s facial region is visible, but whereas E1’s legs are visible and within easy reach of the platform, E2’s arms are within easy reach of her mealworm platform.

Condition 5. (See Figure 2e) E1 covered her mouth and nose with a smaller brown cloth, draping an identical brown cloth over her lap, without occluding limbs or any significant portion of her body. E2 covered her arms with an opaque brown cloth, visually occluding her arms, torso, and shoulders. Both experimenters squatted in the final position. While E1’s facial region is occluded, both her arms and legs are within easy reach of her mealworm platform, where only E2’s legs and facial region are visible and within reach of the platform.

Condition 6. (See Figure 2f) E1 covered her mouth and nose with a smaller brown cloth, draping an identical brown cloth over her lap, without occluding limbs or any significant portion of her body. E2 covered her legs with an opaque brown cloth, visually occluding her legs from the waist down. Both experimenters squatted in the final position. E1 can easily reach her platform with her arms and legs, but has an occluded face, whereas E2’s visible face and arms are within easy reach of her mealworm platform.

### 3.2. Results

Robins reliably approached and retrieved the mealworm from the experimenter standing and uncovered in Condition 1 (binomial probability: *p* < 0.01; see Figure 2a). In Condition 2, subjects selectively chose the mealworm in front of the experimenter who was standing and covered (*p* < 0.05; see Figure 2b). Results from both of these conditions suggest that proximity to the face is a strong factor in decision-making regarding competitive foraging or pilfering and risk-taking (see also Garland et al. 2014). In Condition 3, robins were significantly more likely to selectively approach and retrieve the mealworm from the experimenter covered and squatting (binomial probability: *p* < 0.05; see Figure 2c). Overall, the results of the first three conditions indicate that robins preferentially pilfer prey from human experimenters that are standing, and visually occluded.

Conditions 4, 5, and 6 presented experimenters in which either legs, arms or nose and mouth were visually occluded. Results for all three of these conditions found that robins did not selectively choose either experimenter (binomial probability: *p* = 0.149; see Figure 2d–f).

Taken together, these results indicate that while robins will preferentially pilfer prey from a standing, occluded human, robins do *not* preferentially pilfer near an experimenter with one particular limb region (legs, arms, or nose/mouth) is occluded in comparison with another single region.

## 4. Experiment 2

**Figure 3:**
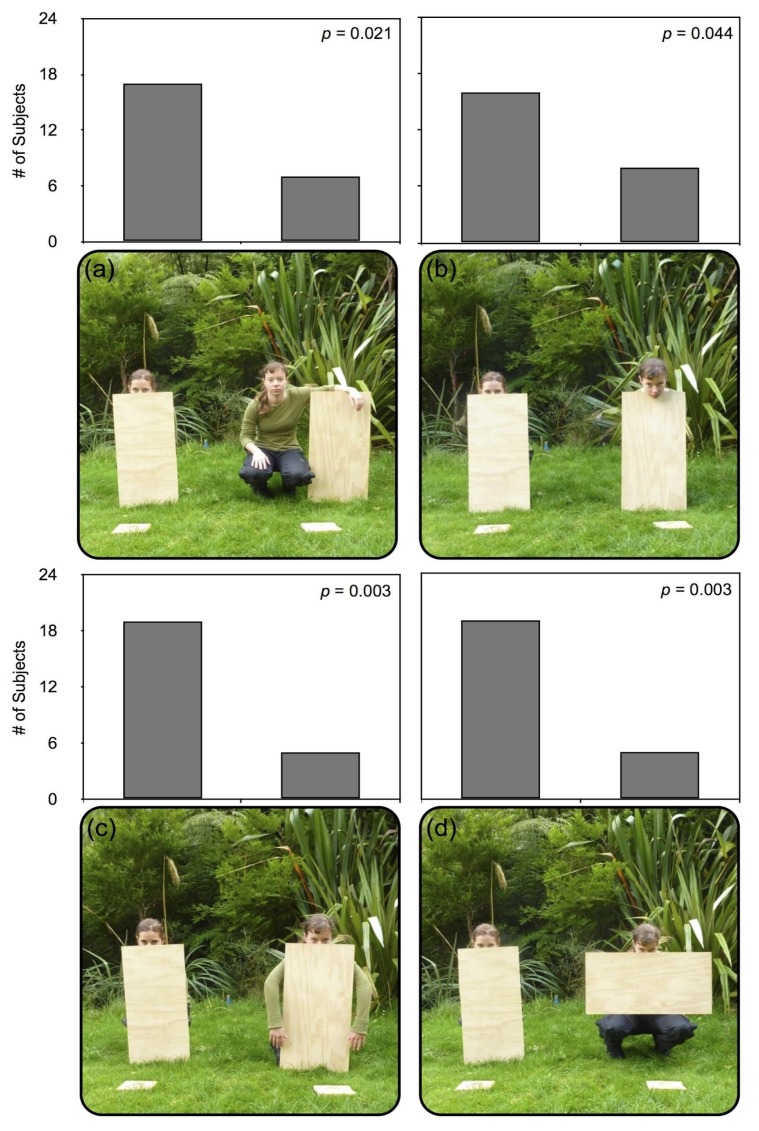
The four conditions presented to robins in Experiment 2, using a wooden plank to obscure the body or limbs of the experimenters (E1 and E2). Results for all four conditions in Experiment 2, displaying the number of subjects pilfering from each experimenter, with one trial per condition per subject. In each condition, all of E1’s limb regions are hidden behind a plank. Condition 1 (**a**) presents E2 with every limb region visible, alongside a plank; Condition 2 (**b**) presents E2 with only the nose/mouth region visible; Condition 3 (**c**) presents E2 with only the arm region visible; Condition 4 (**d**) presents E2 with only the leg region visible.

Experiment 2 alters two fundamental features with comparison to Experiment 1: rather than covering a single “limb region” (both arms, legs, or “beak”) at a time, only one limb region at a time is revealed for comparison, and wooden planks are used to occlude experimenters rather than cloth. In these Conditions, E1 was always entirely occluded. E2 was always fully or partially visible: in Condition 1 she was fully visible (Figure 3a), Condition 2 only her face (including mouth and nose) was visible (Figure 3b), in Condition 3 only her arms were visible (Figure 3c), and in Condition 4 only her legs were visible (Figure 3d). Experimenters were squatting in all conditions. In each condition, E1 squatted behind a wooden plank, her body entirely visually occluded, with her face visible only from just below her eyes (above the bridge of the nose) to the top of her head. E2 varied her position as described in each condition below. Experimenters were counterbalanced between trials.

### 4.1. Procedure

Twenty-four adult robins were exposed to each of four conditions, using Latin Squares to alternate the order of exposure, experimenter side (left and right), and experimenter position (visual access or none) across the 24 robins.

Condition 1. (See Figure 3a) E2 was entirely visible, squatting next to the plank without occluding significant portions of any of her limbs, torso, or face, and could more easily reach her mealworm platform with any of these regions.

Condition 2. (See Figure 3b) E2 squatted behind a wooden plank, her body entirely visually occluded, with her face, including eyes and mouth, entirely visible along the outside edge of the plank, making only her nose and mouth region more visibly within reach of the platform than E1.

Condition 3. (See Figure 3c) E2 squatted behind a wooden plank, with her body occluded and arms visible along the outside edges of the plank, and her face visible only from just below the eyes to the top of her head. E2 was more easily able to access her mealworm plank using only her unoccluded arms.

Condition 4. (See Figure 3d) E2 squatted behind a wooden plank, with her body occluded and legs visible below the long edge of the plank, and her face visible only from just below the eyes to the top of her head. E2 could, therefore, more easily access her mealworm platform with her legs.

### 4.2. Results

In Experiment 2, robins reliably chose the fully occluded experimenter in all four conditions (binomial probability: *p* < 0.05; see Figure 3). Together with the results of Experiment 1, these findings suggest that robins are sensitive to the visibility of all three “limb” regions (legs, arms, nose/mouth), taking this information into account when making decisions about pilfering or foraging activities.

While these results are not directly comparable to primate performance [15], where bait location was also varied and alternately presented as more proximal in distance to either hands or feet, they do suggest that robins possess rudimentary sensitivity to limb visibility within the framework of foraging competition and pilfering.

## 5. Discussion

The present study shows that North Island robins visually attend to human limbs in the context of determining who to steal food from. In most conditions, robins preferred to steal food located near the experimenter whose limbs were obscured by a cloth or board rather than food located near the experimenter whose limbs were not obscured. This is one of the first tests of the role of visual attendance of potential limb availability in a competitive context—stealing food. This may have significant implications for how other species have passed tests designed to examine what individuals understand about the physical acts others are capable of performing.

North Island robins demonstrated significantly different responses in pilfering from human experimenters when a limb region was either visible or obscured, but do not appear to differentiate in preference for a specific region. In Experiment 1, subjects preferred to pilfer from an experimenter who was standing or entirely covered in an opaque cloth (Conditions 1, 2, and 3, see Figure 2a–c), but did not show a preference when only one “limb region” (arms, legs or nose/mouth) was occluded with comparison to another (Conditions 4, 5, and 6, see Figure 2d–f)—so for example, robins did not preferentially avoid unoccluded arms. It is possible that Conditions 2 and 3 were at least partially influenced by wind fluttering the cloth wrapping the standing or crouching experimenter; an unavoidable occurrence in some trials in a peninsular region prone to strong winds, such as Wellington. Regardless, both conditions showed preferential pilfering near the occluded experimenter, despite any avoidance due to wind. In Experiment 2, subjects were consistently more likely to pilfer from an experimenter who was occluded, rather than an experimenter with a limb region showing. Use of a competitive, rather than cooperative, paradigm may have played a role in robins showing a clearer preference than chimpanzees in similar conditions [15], as some evidence has shown that chimpanzees may show more success at cognitive tasks within a competitive framework than a cooperative one [31].

A simple rule for avoiding the human agent with proportionally more limb area visible is certainly one possible explanation for the robins’ response to some conditions. It does not, however, appear to apply proportionally, in which case they should have had a less aversive response in a condition where only the nose/mouth area were exposed (see Figure 3b), presenting far less visible surface area, with comparison to a condition where the entire leg region was exposed (see Figure 3d), presenting far greater visible surface area. There was not a proportionally lower level of avoidance of cases with less body surface area visible than in cases with more, and appear to avoid “beak” and “leg” visible conditions equally. Using the same example, when both experimenters are squatting, robins’ responses also do not appear wholly explained by the proximity of the exposed limb region—even while the exposed leg region was at a closer approximate distance to the prey and the robin than the exposed nose/mouth area, the percentage of responses did not indicate an influence of this difference in distance.

Conversely, in Experiment 1, where experimenters were standing as well as squatting in two conditions, this difference in distance to the head and upper torso in the standing experimenter with comparison to the squatting experimenter may well have a determining factor in the robins’ preference for pilfering from the standing experimenter, whose head and torso were at a greater distance from both the prey and the robin itself. Eyes and gaze do appear to play a role in pilfering in some contexts [32], and proximity to the experimenters’ eyes could certainly have played a role in pilfering preference between standing and squatting positions in the first two conditions of Experiment 1, it does not explain differential results in the remaining conditions where face and eye proximity does not vary between experimenters.

It is important in all of these cases to consider context; even adult robins with frequent exposure to humans traversing their territory are unafraid and seek out, rather than avoid, relatively close proximity to completely unoccluded human bodies. Systematic avoidance of only portions of them therefore, based solely on the proportion showing, would be a surprising behaviour in a species showing little hesitation to approach these same body regions within a meter outside of the context of any experiment obscuring limbs. Such behavioural and ecological contextual details underscore that, while it may be a contributory factor, robins are unlikely to be responding to proportional visibility of humans alone.

This study also does not conclusively indicate that robins perceive a human’s nose and mouth as analogous to beaks, or consider this body region capable of manipulating the environment in the same way. It does, however, suggest that they respond by avoiding this facial region much the same way they do visible legs and arms, even in lieu of observing human behaviour in which the nose/mouth was used in the manipulation of objects or aggression. The birds that took part in these experiments are permanent residents of a fenced sanctuary open to the public, and walking paths transect their territories, inviting regular exposure to human visitors. It is certainly possible that birds in areas of forest less exposed to human visitation, or populations isolated on offshore islands, might show differences in response to the visibility of limb areas.

It has been noted that this species shows potential plasticity in responding to mammalian predators, depending on exposure or lack thereof, and can lose anti-predatory behaviour within a generation of movement to protected areas [32,33]. This suggests the possibility that behavioural responses toward other non-predatory mammals, such as humans, could also be strongly mediated by exposure. New fledglings, within six months of hatching, are often more likely to approach and remain within an extremely close proximity of humans, even in the presence of limb movement, in this more heavily-trafficked area of the forest than birds one year or older (observed in situ). While feeding of the birds is not allowed within the sanctuary, robins in this area are able to readily observe individuals and groups of humans running, walking, pushing prams, and potentially eating as they spend time in the sanctuary, which is also a local tourist attraction. Ecologically, birds (and other species) frequently adapt their behaviour to the physical world around them, from road noise [34], to changes in biodiversity [35]. In this context, it is interesting that the data provided by this study might be indicative that pilfering and foraging responses of a native species could potentially adapt to the novel physical capabilities of an introduced species (mammals). Given the limited adaptation to mammals and lack of clearly defined predator-prey relationship with humans, robins make a particularly interesting model species for such a study and, in particular, allows for testing variability in responses across subjects with differing amounts of exposure to human activity (with and without paths intersecting territories, differing distances from the sanctuary entrance, populations on inaccessible offshore islands, etc.).

A variety of species have been tested on their reaction to the physical properties of objects and their interaction with other objects and spaces around them, displaying varying levels of complexity and success. Previous experimentation has shown that nonhuman animals, both mammals [36,37,38,39] and birds [40,41,42], represent and reason about physical objects. Physical contact in particular is a salient cue in physics problems, and while it is to some extent attended to by chimpanzees [43], for example, bonobos and rooks appear to have more difficulty [44]. A study with chicks (*Gallus gallus*) [45] found that they showed intuitive reasoning about occluded objects. After imprinting upon a specific object as a social partner upon hatching, chicks were given a choice between a screen leaned at an angle that was consistent with that same object being hidden beneath it, or one leaning at such an angle that the object could not be hidden behind it. Chicks consistently chose the screen angled such that their imprinted “companion” could have been hidden beneath it, indicating a rudimentary framework encompassing physical properties of the objects around them. Exact mechanisms and whether such understandings of folk physics is due to causal relations [40] or by trial and error [43], either in specific contexts, or when reasoning about physical interaction altogether, is still largely unclear. Whether such an understanding could be extended to physical properties and limitations as it relates to other individuals’ bodies is a question yet unexplored in most species [15] (Vonk and Subiaul, 2009) that we begin to address here in New Zealand robins.

The present study demonstrates that a largely biologically naïve bird has the ability to form rules about a physiologically dissimilar species based on observable features—but does not answer whether they are generalizable—although we begin to disentangle some potentially relevant and irrelevant visual cues [15]. If a mealworm was within reach only of an experimenter’s legs, but not arms, for example, would robins adapt their response? Given the vast differences in avian and primate physical structure and use of limbs, such a question is even more complex when asked inter- than intraspecies, and of a naïve bird species in reference to humans. Vonk and Subiaul [15] raise the question of whether causal reasoning is specific to social reasoning, and whether this might be the reason chimpanzees were unable to generalise the physical causality of limbs for humans. That cannot entirely explain the results of either Vonk and Subiaul’s or the present study, but makes it all the more interesting that these birds are able to show even a basic systematic response despite the lack of co-evolution or clear ecological roles (e.g., predatory). Whether this is due to an underlying mechanism or exposure to humans walking around the sanctuary paths through robin territories is still an open question, begging further investigation with variably-exposed populations.

In wild animals, exposure and environmental conditions may play a significant role in how such cognitive abilities are demonstrated in context—even if some shared representations exist across species, responses between species based on differing physiology (a raptor’s acute visual system for example) can still be acquired and adapted to through exposure to those species. For example, New Caledonian crows show a context-dependent use of tools for foraging [46,47,48,49,50] by manipulating wire to form a hook or choosing the right length or type of tool to fit a specific task. Mixed results show that, given the right conditions, wolves outperform dogs in recognising distal pointing [51,52,53].

A foundational understanding of physical capability underlying reasoning about object-directed action [8] might not be specific to within the primate order. While certain features may well be primate-specific, a basic framework of observable physical cues and their relation to an agent’s physical capability could be a more broadly applicable capacity. Evidence of ravens (*Corvus corax*) using referential declarative signals amongst themselves in the wild, offering non-edible items in specific social contexts [54], or self-agency in chimpanzees [55], recognising abstract cursor control without direct physical contact may both be seen as related to such a capacity, though the exact extent remains to be seen.

The present study lays the groundwork for further investigation; whether North Island robins can flexibly apply visual information that may be salient to physical capability (i.e., limb visibility and distance to an object), and to what extent the mobility of the visible part of the body influences response (limb versus torso) is certainly an aim for future exploration. Using methodology such as that presented here to determine the role of competition and limb visibility/availability within, and between, species in food access and decision-making paradigms may shed light on success and failure of a variety of species at tasks aimed at understanding perception of causality and intention. It also opens the door to a potentially closer examination of changes in such behaviours over the lifespan of the animal. This is the first study to look at what cues a wild population responds to within the context of human physical capability and limb visibility with such broad morphological differences as exist between humans and songbirds, and within a natural, spontaneous context in the wild. Adapting experiments that closely examine behavioural responses to reasoning about physical causality to a natural setting, with less-studied species adds valuable data about the form and function of cognition across species in an ecologically relevant context.

## 6. Conclusions

Wild North Island robins (*Petroica longipes*) attend visually to human limbs when choosing who to steal food from. The present study shows that in most conditions, robins preferred to steal food located near an experimenter whose limbs were obscured by a cloth or board rather than food located near an experimenter whose limbs were clearly visible. Our results may have significant implications for how other species have passed tests designed to examine what individuals understand about the physical acts others are capable of performing.
